# Subgrouping of Japanese middle-aged women attending a menopause clinic using physical and psychological symptom profiles: a cross-sectional study

**DOI:** 10.1186/s12905-014-0148-z

**Published:** 2014-11-25

**Authors:** Masakazu Terauchi, Asuka Hirose, Mihoko Akiyoshi, Yoko Owa, Kiyoko Kato, Toshiro Kubota

**Affiliations:** Department of Women’s Health, Tokyo Medical and Dental University, Yushima 1-5-45, Bunkyo, Tokyo 113-8510 Japan; Department of Obstetrics and Gynecology, Tokyo Medical and Dental University, Yushima 1-5-45, Bunkyo, Tokyo 113-8510 Japan

**Keywords:** Principal component analysis, Cluster analysis, Musculoskeletal pains, Tiredness, Vasomotor symptoms, Depression, Anxiety, Insomnia

## Abstract

**Background:**

Women in the menopausal transition and the postmenopausal period are affected with vasomotor symptoms, urogenital atrophy, sexual dysfunction, somatic symptoms, cognitive difficulty, sleep disturbance, and psychological problems. It is important to gain a better understanding of the complexity and diversity of climacteric disturbance in order to optimize treatments for individual patients. The aim of this study was to identify subgroups of Japanese perimenopausal and postmenopausal women attending a menopause clinic based on their physical and psychological symptom profiles.

**Methods:**

We administered the Menopausal Health-Related Quality of Life questionnaire to 491 Japanese women aged 40–64 years who had enrolled in the Systematic Health and Nutrition Education Program at the Menopause Clinic of the Tokyo Medical and Dental University Hospital between 2005 and 2012. We performed a principal component analysis followed by a hierarchical cluster analysis of the responses to 9 physical and 12 psychological items on the questionnaire.

**Results:**

The first analysis extracted 3 principal components that defined the variance of physical and psychological symptom profiles: depression, somatic, and vasomotor/sleep. A subsequent cluster analysis was performed based on the 3 principal components to generate 4 clusters, CL8 (N = 162; 33.0%), CL6 (N = 111; 22.6%), CL5 (N = 102; 20.8%), and CL4 (N = 116; 23.6%). CL8 included women who only had mild-to-moderate musculoskeletal pains and tiredness. All women in CL6, CL5, and CL4 described their musculoskeletal pains and tiredness as moderate to severe. The women in CL5 also had moderate-to-severe vasomotor symptoms, while the women in CL4 also suffered from moderate-to-severe psychological symptoms, such as depression, anxiety, and insomnia.

**Conclusions:**

Distinct subgroups of Japanese perimenopausal and postmenopausal women were identified based on their symptom profiles. Menopausal symptoms were shown to accumulate in this population in the order of musculoskeletal pains and tiredness, vasomotor symptoms, and psychological symptoms.

## Background

Women in the menopausal transition and the postmenopausal period are affected with vasomotor symptoms, urogenital atrophy, sexual dysfunction, somatic symptoms, cognitive difficulty, sleep disturbance, and psychological problems. Some of these effects, particularly vasomotor symptoms and urogenital atrophy, are closely associated with estrogen deficiency, whereas the exact mechanism underlying the other symptoms is not fully understood.

Therefore, it is important to gain a better understanding of the complexity and diversity of climacteric disturbance in order to optimize treatments for individual patients. Accordingly, previous trials have categorized menopausal symptoms of non-menopause clinic samples [1] and population-based samples [2–8], and identified subgroups of menopausal women on the basis of their responses to symptom checklists. Yet, subgrouping of menopause clinic samples, which would be most relevant to clinical practice, has not been reported.

The aim of the present study was to identify subgroups of Japanese perimenopausal and postmenopausal women attending a menopause clinic based on their physical and psychological symptom profiles.

## Methods

In this cross-sectional study, we examined the medical records of a study population similar to that analyzed in our previous studies [9–15]. Specifically, we retrospectively analyzed the first-visit records of 491 Japanese perimenopausal and postmenopausal women aged 40–64 years who had enrolled in the Systematic Health and Nutrition Education Program conducted at the Menopause Clinic of the Tokyo Medical and Dental University Hospital between 2005 and 2012. All middle-aged women who had enrolled in this program were referred to our clinic for the treatment of their menopausal symptoms, and all participants provided informed consent. Before beginning our investigation, we obtained approval for the study protocol from the Tokyo Medical and Dental University Review Board and confirmed that all participants provided informed consent. We ensured that all study procedures were implemented in accordance with the Declaration of Helsinki.

A woman was defined as being in the menopausal transition if she menstruated within the past 12 months but had either missed a period or had experienced irregular cycles in the past 3 months; a woman was defined as being postmenopausal if she had not menstruated in the past 12 months [16]. At their initial visit, women were interviewed by physicians and nutritionists, and they provided data regarding their menopausal symptoms and quality of life during the past month by completing the Menopausal Health-Related Quality of Life questionnaire, which we developed and validated at our clinic [9–15,17]. The Menopausal Health-Related Quality of Life questionnaire is a modification of the Women’s Health Questionnaire developed by Hunter et al. and contains 38 items scored on a 4-point or binary scale covering 4 major domains (physical health, mental health, life satisfaction, and social involvement) of a woman’s health during the menopausal transition [1,18].

The items used to assess the 2 domains of interest in the current study, physical health and mental health, are shown in Table 1. The physical health domain comprised 9 items that assessed somatic and vasomotor symptoms, and the mental health domain comprised 12 items that assessed depressed mood, cognitive difficulties, anxiety and fears, sexual functioning, and sleep problems. For convenience, the scoring system shown in Table 1 is in the reverse order to that used in the quality of life questionnaire in our clinical practice; higher scores indicate worse physical and mental functioning.

**Table 1:** **Physical and mental health domain items in the Menopausal Health-Related Quality of Life questionnaire**

	**0–1 times a month**	**1–2 times a week**	**3–4 times per week**	**Almost every day**
	**(** ***none*** **)**	**(** ***mild*** **)**	**(** ***moderate*** **)**	**(** ***severe*** **)**
***Physical health domain***				
**Nausea**	0	1	2	3
**Dizziness**	0	1	2	3
**Numbness**	0	1	2	3
**Muscle and joint pains**	0	1	2	3
**Tiredness**	0	1	2	3
**Headaches**	0	1	2	3
**Frequent urination**	0	1	2	3
**Hot flush**	0	1	2	3
**Night sweats**	0	1	2	3
***Mental health domain***				
**Loss of interest in things**	0	1	2	3
**Lack of enjoyment**	0	1	2	3
**Low energy**	0	1	2	3
**Depressed mood**	0	1	2	3
**Poor memory**	0	1	2	3
**Difficulty in concentration**	0	1	2	3
**Frightened/panicky feelings**	0	1	2	3
**Feel tense/wound up**	0	1	2	3
**Dissatisfaction with sexual relationship**	0	1	2	3
**Difficulty in initiating sleep**	0	1	2	3
**Non-restorative sleep**	0	1	2	3
**Low self-esteem**	0	1	2	3

The symptom score profiles of participants were subjected to a principal component analysis (PCA) to examine the relationships among the 21 physical and mental symptoms and to define factors (principal components [PCs]), composed of scores of related symptoms, that could account for most of the variance in profiles of symptom scores. The appropriate number of PCs was determined from the point on the Scree plot where eigenvalues leveled off. PC values for each woman were then calculated based on the formulas obtained from the PCA.

Subsequently, a hierarchical cluster analysis was performed using Ward’s minimum variance technique to subgroup women based on the Euclidean distances among the PC values.

All statistical analyses were performed with SAS 9.2 (SAS Institute, Cary, NC, USA). *P* <0.05 was defined as statistically significant.

## Results

A total of 491 women were included in the study, and the mean ± standard deviation age of participants was 51.8 ± 5.4 years; 44% of participants were classified as being in the menopausal transition, and 56% were classified as postmenopausal. The eigenvalues, percent of variance, and factor loading (eigenvectors) obtained from the initial PCA of study participants’ symptom score profiles are shown in Table 2. Three PCs were identified, accounting for 48% of variance. The first PC (PC1) accounted for 32.8% of variance, for which the 3 symptom scores with largest eigenvectors were low energy, depressed mood, and lack of enjoyment. Therefore, PC1 was best characterized as the component for “depression”. Likewise, PC2, accounting for 9.1% of variance, was characterized as “somatic” because its top 3 symptoms were dizziness, muscle and joint pain, and nausea. PC3, which accounted for 6.1% of variance, was characterized as “vasomotor/sleep”, as symptoms included night sweats, difficulty in initiating sleep, and hot flush. Based on the PCA, PC1, 2, and 3 for each woman was calculated as follows:

**Table 2:** **Principal component analysis**

	**Eigenvalue**	**% of variance**	**Eigenvector**
*Principal component 1:*	6.89	32.8	
*“depression”*			
Low energy			0.321
Depressed mood			0.318
Lack of enjoyment			0.302
*Principal component 2:*	1.91	9.1	
*“somatic”*			
Dizziness			0.371
Muscle and joint pains			0.320
Nausea			0.318
*Principal component 3:*	1.28	6.1	
*“vasomotor/sleep”*			
Night sweats			0.434
Difficulty in initiating sleep			0.318
Hot flush			0.317

- $$ \mathrm{P}\mathrm{C}1 = 0.321\ *\ ``\mathrm{low}\ \mathrm{energy}"\ \mathrm{score} + 0.318\ *\ ``\mathrm{depressed}\ \mathrm{mood}"\ \mathrm{score} + 0.302\ *\ ``\mathrm{lack}\ \mathrm{of}\ \mathrm{enjoyment}"\ \mathrm{score} $$
- $$ \mathrm{P}\mathrm{C}2 = 0.371\ *\ ``\mathrm{dizziness}"\ \mathrm{score} + 0.320\ *\ ``\mathrm{muscle}\ \mathrm{and}\ \mathrm{joint}\ \mathrm{pains}"\ \mathrm{score} + 0.318\ *\ ``\mathrm{nausea}"\ \mathrm{score} $$
- $$ \mathrm{P}\mathrm{C}3 = 0.434\ *\ ``\mathrm{night}\ \mathrm{sweats}"\ \mathrm{score} + 0.318\ *\ ``\mathrm{difficulty}\ \mathrm{in}\ \mathrm{in}\mathrm{itiating}\ \mathrm{sleep}"\ \mathrm{score} + 0.317\ *\ ``\mathrm{hot}\ \mathrm{flush}"\ \mathrm{score} $$

**Figure 1:**
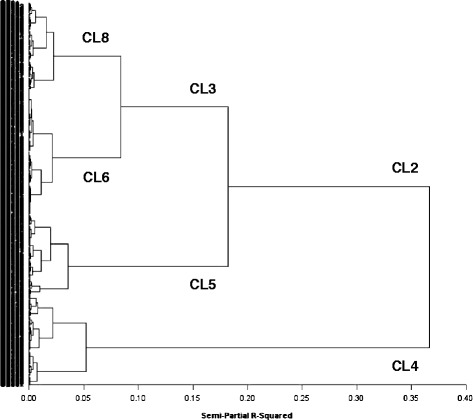
**Cluster analysis.** Four distinct clusters (CLs) were obtained from the subsequent cluster analysis based on the Euclidean distances among the PC values: CL8 (N = 162; 33.0%), CL6 (N = 111; 22.6%), CL5 (N = 102; 20.8%), and CL4 (N = 116; 23.6%).

Four distinct clusters (CLs) were obtained from the subsequent cluster analysis based on the Euclidean distances among the PC values: CL8 (N =162; 33.0%), CL6 (N = 111; 22.6%), CL5 (N = 102; 20.8%), and CL4 (N = 116; 23.6%) (Figure 1).

Baseline physical characteristics of the study participants by cluster are shown in Table 3. One-way analysis of variance and a chi-square test showed that height, waist-hip ratio, systolic/diastolic pressure, pulse rate, and the proportions of women who smoked and exercised regularly differed among the groups. The average physical and psychological symptom scores of the 4 clusters are shown in Table 4. The Kruskal-Wallis test showed that scores for every item were significantly different among the groups. On the basis of the average score for each item, the 4 clusters were characterized as follows: CL8 was composed of women who only had mild-to-moderate muscle and joint pain and tiredness. All the women in CL6, CL5, and CL4 assessed their musculoskeletal pains and tiredness as moderate to severe. The women in CL5 also had moderate-to-severe vasomotor symptoms, while the women in CL4 suffered from moderate-to-severe psychological symptoms, such as depression, anxiety, and insomnia.

**Table 3:** **Baseline physical characteristics of the study participants (N = 491)**

	**CL8**	**CL6**	**CL5**	**CL4**	***P***
	**(N = 162)**	**(N = 111)**	**(N = 102)**	**(N = 116)**	
Age (years)	52.1 ± 6.0	51.0 ± 5.5	51.5 ± 5.1	52.5 ± 4.8	0.205
Perimenopausal/postmenopausal (%)	40.3/59.7	49.1/50.9	46.5/53.5	41.4/58.6	0.455
Height (cm)	156.4 ± 5.6	158.1 ± 5.3	157.1 ± 5.4	155.5 ± 5.4	0.004
Body weight (kg)	53.0 ± 8.5	53.0 ± 8.7	54.1 ± 9.9	51.9 ± 9.4	0.337
Body mass index (kg · m^–2^)	21.6 ± 3.3	21.2 ± 3.3	21.9 ± 3.7	21.4 ± 3.6	0.491
Body fat (%)	26.7 ± 7.3	26.9 ± 8.4	27.6 ± 7.8	26.4 ± 7.8	0.806
Muscle mass (kg)	35.9 ± 2.7	36.0 ± 2.8	36.2 ± 3.4	35.3 ± 3.0	0.220
Water mass (kg)	27.5 ± 2.6	27.3 ± 2.9	27.8 ± 3.5	26.9 ± 2.8	0.211
Waist circumference (cm)	78.8 ± 9.0	77.2 ± 9.5	80.1 ± 9.8	77.6 ± 10.2	0.516
Hip circumference (cm)	92.0 ± 5.8	91.1 ± 6.2	91.5 ± 6.1	90.5 ± 7.2	0.295
Waist-hip ratio (%)	85.5 ± 6.0	84.5 ± 6.3	87.4 ± 6.4	85.6 ± 6.8	0.013
Systolic pressure (mmHg)	122.2 ± 14.5	124.1 ± 17.9	126.8 ± 14.9	127.7 ± 16.8	0.021
Diastolic pressure (mmHg)	78.5 ± 10.1	78.6 ± 12.2	81.8 ± 11.0	82.3 ± 11.6	0.007
Pulse rate (min^–1^)	61.7 ± 9.6	62.2 ± 8.0	64.2 ± 10.9	65.4 ± 12.6	0.015
Cardio-ankle vascular index	7.5 ± 0.8	7.5 ± 0.8	7.5 ± 0.8	7.5 ± 0.7	0.944
Daily alcohol consumption (%)	8.6	10.9	17.5	10.6	0.184
Smoking (%)	6.6	12.7	17.2	15.9	0.046
Regular exercise (%)	52.2	39.4	51.0	27.8	< 0.001

Data are expressed as mean ± standard deviation.

Statistical analyses were performed using one-way analysis of variance and chi-square tests.

**Table 4:** **Average physical and psychological symptom scores of the 4 clusters (N = 491)**

	**CL8**	**CL6**	**CL5**	**CL4**	***P***
	**(N = 162)**	**(N = 111)**	**(N = 102)**	**(N = 116)**	
*Physical health domain*					
Nausea	0.0 ± 0.2	0.3 ± 0.7	0.5 ± 1.0	0.6 ± 1.0	< 0.0001
Dizziness	0.2 ± 0.5	0.7 ± 0.9	0.6 ± 1.0	0.7 ± 1.0	< 0.0001
Numbness	0.4 ± 1.0	0.8 ± 1.1	0.8 ± 1.2	1.1 ± 1.3	< 0.0001
Muscle and joint pains	1.6 ± 1.2	2.3 ± 1.0	2.2 ± 1.1	2.3 ± 1.1	< 0.0001
Tiredness	1.0 ± 1.0	2.2 ± 0.9	2.0 ± 1.1	2.6 ± 0.9	< 0.0001
Headaches	0.4 ± 0.7	0.8 ± 1.0	1.0 ± 1.1	1.3 ± 1.2	< 0.0001
Frequent Urination	0.6 ± 1.0	0.9 ± 1.2	1.3 ± 1.3	1.3 ± 1.4	< 0.0001
Hot flush	0.5 ± 0.9	0.6 ± 0.9	2.1 ± 1.1	1.4 ± 1.2	< 0.0001
Night sweats	0.1 ± 0.4	0.2 ± 0.5	2.0 ± 1.1	1.3 ± 1.2	< 0.0001
*Mental health domain*					
Loss of interest in things	0.1 ± 0.4	0.7 ± 0.9	0.7 ± 0.8	2.2 ± 1.1	< 0.0001
Lack of enjoyment	0.0 ± 0.2	0.8 ± 0.7	0.5 ± 0.6	2.6 ± 0.8	< 0.0001
Low energy	0.1 ± 0.3	1.3 ± 0.7	0.8 ± 0.8	2.8 ± 0.5	< 0.0001
Depressed mood	0.2 ± 0.4	1.5 ± 0.8	0.9 ± 0.8	2.9 ± 0.4	< 0.0001
Poor memory	0.8 ± 0.9	1.3 ± 1.0	1.3 ± 1.0	1.7 ± 1.1	< 0.0001
Difficulty in concentration	0.8 ± 0.9	1.3 ± 1.0	1.3 ± 1.0	1.7 ± 1.1	< 0.0001
Frightened/panicky feelings	0.3 ± 0.6	1.3 ± 1.0	1.1 ± 0.9	2.2 ± 1.0	< 0.0001
Feel tense/wound up	0.3 ± 0.6	0.8 ± 1.0	0.9 ± 1.1	1.8 ± 1.2	< 0.0001
Dissatisfaction with sexual relationship	0.1 ± 0.5	0.3 ± 0.7	0.4 ± 0.9	0.4 ± 0.9	0.0134
Difficulty in initiating sleep	0.4 ± 0.7	0.8 ± 1.0	1.6 ± 1.2	1.7 ± 1.3	< 0.0001
Non-restorative sleep	0.5 ± 0.9	1.3 ± 1.2	1.7 ± 1.2	2.0 ± 1.1	< 0.0001
Low self-esteem	0.5 ± 0.8	1.4 ± 1.2	1.1 ± 1.1	2.1 ± 1.2	< 0.0001

Data are expressed as mean ± standard deviation.

Statistical analyses were performed using the Kruskal-Wallis test.

Women with more severe symptoms tended to have higher systolic/diastolic pressure, higher pulse rates, smoked more, and exercised less frequently than women with milder symptoms (Table 3).

## Discussion

In this study, we analyzed the responses to 9 physical and 12 psychological items on the Menopausal Health-Related Quality of Life questionnaire by 491 Japanese women attending our menopause clinic aged 40–64 years. The first analysis extracted 3 PCs (depression, somatic, and vasomotor/sleep) that defined the variance of physical and psychological symptom profiles of the women. A subsequent cluster analysis was performed based on the 3 PCs to generate 4 clusters: CL8, CL6, CL5, and CL4. CL8 was composed of women who had only mild-to-moderate musculoskeletal pains and tiredness. All women in CL6, CL5, and CL4 described their musculoskeletal pains and tiredness as moderate to severe. The women in CL5 also had moderate-to-severe vasomotor symptoms, while the women in CL4 also suffered from moderate-to-severe psychological symptoms, such as depression, anxiety, and insomnia.

Many previous studies have categorized menopausal symptoms of non-menopause clinic samples and population based samples, and identified subgroups of women in the menopausal transition on the basis of their responses to symptom checklists. In 1986, Hunter et al. first introduced their “Women’s Health Questionnaire”, and they performed a PCA of responses to the 36-item symptom checklist among 682 English women attending an ovarian screening program aged 45–65 years [1]. Based on the analysis, they defined 9 symptom clusters, namely “somatic symptoms”, “depressed mood”, “cognitive difficulties”, “anxiety/fears”, “sexual functioning”, “vasomotor symptoms”, “sleep problems”, “menstrual”, and “attractive”.

Similar studies using PCA methods have been performed to identify menopausal symptom clusters specific to Hong Kong Chinese [2] and Taiwanese [3]; to examine cross-cultural variation among the United States, Spain, Lebanon, and Morocco [4]; to investigate how symptoms cluster across menopausal transition stages [5]; and to determine which symptom cluster has the greatest effect on quality of life [6]. In a random telephone survey of Hong Kong Chinese women, Ho et al. identified 5 symptom clusters: psychological, musculoskeletal/gastrointestinal, non-specific somatic, respiratory, and vasomotor [2]; in a resident cohort study of Taiwanese women, Fuh et al. identified 4 symptom clusters: musculoskeletal, non-specific somatic complaints, urogenital, and vasomotor [3]. Sievert et al. showed in their study of general population that the inter-correlation among symptoms differed in country-specific ways; for example, hot flush grouped with vaginal dryness and sexual symptoms in Spain, with general somatic symptoms in Morocco, and did not cluster with other symptoms in the United States or Lebanon [4]. Based on the observations from the Seattle Midlife Women’s Health Study (SMWHS), Cray et al. revealed similar factor structures across the 4 menopausal transition stages (late reproductive, early or late menopausal transition, or early postmenopause) in that each stage revealed a mood component, a vasomotor component, and a pain component [5]. A study by Greenblum et al. identified 3 symptom clusters in middle-aged Floridian community-dwelling women: (1) anxiety, irritability, and fatigue; (2) weight gain and urinary stress incontinence; and (3) vaginal dryness and sleep disturbances, which had the greatest impact on quality of life [6].

A different approach using cluster analysis was taken to investigate the relationships between patterns of depressed mood and menopausal transition stages [19] or to identify clusters of female urological symptoms [20]. Using the data from SMWHS, Woods et al. revealed that the majority of women experienced the menopausal transition without a high severity of depressed mood, while a small group of women had mood worsening over time and others improved [19]. Hall et al. conducted a cluster analysis of urological symptoms among women in the Boston Area Community Health Survey and identified 4 clusters, which were distinguished by the severity of storage symptoms, frequency symptoms, urinary incontinence, and interference [20].

Recently, Cray et al. introduced latent class analysis to identify subgroups of women in the menopausal transition stage that experienced the same cluster of representative symptoms [7,8]. They first selected 5 symptoms based on PCA of the SMWHS participants’ responses to the 47-item symptom checklist: sleep, pain, mood, cognitive, and tension. Multilevel latent class analysis using scores for hot flush and these 5 symptoms identified 3 classes: low severity levels for all symptoms; low severity hot flush and moderate severity levels for all other symptom factors; high severity hot flush with lower severity levels of all other symptom factors. In their most recent report, Woods et al. identified several endocrine biomarkers associated with the distinction among the subgroups, including estrogen, follicle stimulating hormone, epinephrine, and norepinephrine [21].

Similar to our findings, a 2013 SMWHS report by Cray et al. identified 3 major components of symptoms identified by PCA [5]. These included psychological, vasomotor, and musculoskeletal pains, of which the psychological component accounted for more than 30% of variance of the profile of menopausal symptoms. Cross-cultural diversity in menopausal symptomatology has been frequently demonstrated [22], and fewer Asian women have been shown to report vasomotor and other menopausal symptoms compared with their Caucasoid counterparts [23]; nevertheless, these fundamental components might be similar between the United States and Japan.

One of the major limitations of our study was that the participants were recruited in a clinical setting, and the results were not necessarily representative of the general population of Japan. Nevertheless, the findings of the current study are relevant to clinical practice as they address the complex symptomatology in women attending a menopause clinic.

## Conclusions

In conclusion, distinct subgroups of Japanese perimenopausal and postmenopausal women were identified based on the severity of depression, somatic, and vasomotor/sleep symptoms. This subgrouping of women revealed that menopausal symptoms accumulate in this population in the order of musculoskeletal pains, vasomotor symptoms, and psychological symptoms.
